# Identifying patients with malignant spinal cord compression (MSCC) near end of life who can benefit from palliative radiotherapy

**DOI:** 10.1186/s13014-022-02117-z

**Published:** 2022-08-17

**Authors:** Dirk Rades, Barbara Segedin, Steven E. Schild, Darejan Lomidze, Theo Veninga, Jon Cacicedo

**Affiliations:** 1 Department of Radiation Oncology, University of Lübeck, Ratzeburger Allee 160, 23562 Lübeck, Germany; 2 Department of Radiotherapy, Institute of Oncology Ljubljana and University of Ljubljana, Ljubljana, Slovenia; 3 Department of Radiation Oncology, Mayo Clinic Scottsdale, Scottsdale, AZ USA; 4 Radiation Oncology Department, Ingorokva High Medical Technology University Clinic and Tbilisi State Medical Univiversity, Tbilisi, Georgia; 5 Department of Radiotherapy, Dr. Bernard Verbeeten Institute, Tilburg, The Netherlands; 6 Department of Radiation Oncology, Biocruces Bizkaia Health Research Institute and Cruces University Hospital, Barakaldo, Vizcaya Spain

**Keywords:** Metastatic spinal cord compression, End of life, Palliative radiotherapy, Functional outcomes, Prognostic score

## Abstract

**Background:**

A previous score predicted death ≤ 2 months following radiotherapy for MSCC. For patients with a high probability of early death, best supportive care was recommended. However, some of these patients may benefit from radiotherapy regarding preservation or improvement of motor function. To identify these patients, an additional score was developed.

**Methods:**

Pre-treatment factors plus radiotherapy regimen were retrospectively evaluated for successful treatment (improved motor function or remaining ambulatory without aid) and post-treatment ambulatory status in 545 patients who died ≤ 2 months. Factors included age, interval from tumor diagnosis until MSCC, visceral metastases, further bone metastases, primary tumor type, sex, time developing motor deficits, pre-treatment ambulatory status, and number of affected vertebrae. Factors significant on both multivariable analyses were included in the score (worse outcomes 0 points, better outcomes 1 point).

**Results:**

On multivariable analyses, myeloma/lymphoma, time developing motor deficits > 14 days, and pre-treatment ambulatory status were significantly associated with both successful treatment and ambulatory status, affection of 1–2 vertebrae with successful treatment only. On univariable analyses, 1 × 8 and 5 × 4 Gy were not inferior to 5 × 5 Gy and longer-course regimens. Considering the three factors significant for both endpoints, three groups were designed (0, 1, 2–3 points) with treatment success rates of 4%, 15% and 39%, respectively (*p* < 0.0001), and post-treatment ambulatory rates of 4%, 43% and 86%, respectively (*p* < 0.0001).

**Conclusion:**

This score helps identify patients with MSCC who appear to benefit from palliative radiotherapy in terms of improved motor function or remaining ambulatory in spite of being near end of life.

## Background

Metastatic spinal cord compression (MSCC) is a serious condition that occurs in 5–10% of cancer patients [1, 2]. Radiotherapy alone has been used for the vast majority of patients with MSCC until 2005, when a randomized trial showed that treatment outcomes were significantly improved in selected patients with the addition of upfront decompressive surgery [3]. However, this trial was limited to patients with a good performance status and an expected survival time of at least 3 months. Thus, patients with survival prognoses of ≤ 2 months remain candidates for radiotherapy alone. However, considering the very short remaining lifespan of these patients, one may question whether they really benefit from radiation treatment or should receive best supportive care (BSC) instead?

In 2013, a scoring tool was developed in order to identify patients with MSCC dying within 2 months after treatment [4]. This tool considered seven independent predictors of survival, namely performance score, primary tumor type, other bone metastases, visceral metastases, interval from tumor diagnosis to MSCC and time developing motor deficits prior to radiotherapy. Scoring points ranged from 11 to 25 points. For patients with a high probability of dying within 2 months, BSC alone was recommended. This was done because it was thought that the benefit of treatment would be short lived. However, on further reflection, some of these patients may benefit from palliative radiotherapy in terms of preservation or improvement of their motor function and ambulatory status, despite their very limited survival prognoses. To identify these patients, the current study was performed that included only patients with MSCC who died within 2 months following treatment. The major goal of this study is to develop an additional score for estimating the probability of a successful radiation treatment, defined as improved motor function or remaining ambulatory without aid, and the probability of being ambulatory after radiotherapy.

## Patients and methods

In a database of 2,610 patients irradiated for MSCC between 1992 and 2021, 545 patients were identified who died within 2 months following treatment. These patients were included in the present retrospective study that achieved approval from the Ethics Committee at the University of Lübeck (reference number 22-194). For simplification, the term MSCC included both spinal cord compression caused by vertebral metastases from solid tumors and spinal cord compression from hematologic malignancies (myeloma or lymphoma). Pre-treatment factors were evaluated for associations with successful treatment (improved motor function or remaining ambulatory without aid) and post-treatment ambulatory status. Improvement of motor function was defined as a change by at least one point (category) on a 4-point scale modified according to Tomita et al. (1 ambulatory without aid, 2 ambulatory with aid, 3 not ambulatory, 4 complete paraplegia) [5].

These factors included age at the start of radiotherapy (≤ 65 vs. > 65 years, median age: 65 years), interval from tumor diagnosis until MSCC (≤ 15 vs. > 15 months [4, 6]), visceral metastases at the start of radiotherapy (no vs. yes), further bone metastases at the start of radiotherapy (no vs. yes), primary tumor type (breast cancer vs. prostate cancer vs. myeloma/lymphoma vs. lung cancer vs. other malignancies), sex (female vs. male), time developing motor deficits prior to radiotherapy (0–7 vs. 8–14 vs. > 14 days [6, 7]), pre-treatment ambulatory status (no vs. yes), and number of vertebrae affected by MSCC (1–2 vs. ≥ 3 [6]). The Eastern Cooperative Oncology Group (ECOG) performance score was not included, because this score and pre-treatment ambulatory status were confounding variables (non-ambulatory patients had an ECOG score of 3–4, and most ambulatory patients an ECOG score of 1–2). Factors that were significantly associated with treatment success and post-treatment ambulatory status on univariable and multivariable analyses were included in the scoring tool (worse outcomes = 0 points, better outcomes = 1 point). In addition, the radiotherapy regimen was investigated with respect to treatment success and post-treatment ambulatory status. 1 × 8 Gy and 5 × 4 Gy [equivalent doses in 2 Gy fractions (EQD2) using an α/β ratio of 10 Gy = 12.0 Gy and 23.3 Gy, respectively] were compared to 5 × 5 Gy (EQD2 = 31.25 Gy) and longer-course regimens with 30–42 Gy in 10–20 fractions (EQD2 = 32.5–43.2 Gy) [8, 9].

Moreover, pre-treatment factors and radiotherapy regimen were evaluated with respect to regaining ambulatory status (in the 375 non-ambulatory patients) and maintaining ambulatory status (in the 170 ambulatory patients). Since pre-treatment ambulatory status could not be evaluated in these additional analyses, it was replaced by the ECOG performance score (1–2 vs. 3–4 [6, 7]).

### Statistical analyses

The univariable analyses were performed with the Chi-square test or the Fisher’s exact test (for n < 5 in at least one cell). When applying the Bonferroni adjustment for 10 tests, *p* values of < 0.005 were considered significant representing an alpha level of < 0.05. *p* values < 0.07 were considered indicating a trend. For multivariable analysis, logistic regression models were fitted. Initially, all pre-treatment variables that achieved significance or showed a trend on univariable analyses were included. A backward elimination was applied (with a 0.10 significance level for removal from the model) to reach the final parsimonious model. Statistical analyses were done with SAS 9.4 (SAS Institute Inc, Cary, NC, USA).

## Results

On univariable analyses, successful treatment was significantly associated with favorable tumor type (myeloma/lymphoma, *p* < 0.0001), time developing motor deficits > 14 days (*p* < 0.0001), pre-treatment ambulatory status (*p* < 0.0001), affection of 1–2 vertebrae (*p* < 0.001) (Table 1). In addition, absence of visceral metastases showed a trend (*p* = 0.066). In the subsequent multivariable analysis, primary tumor type [odds ratio (OR) 7.47, 95% confidence interval (CI) 2.60–21.46, *p* = 0.004), time developing motor deficits (OR 5.86, 95% CI 2.94–11.67, *p* < 0.0001], pre-treatment ambulatory status (OR 2.10, 95% CI 1.16–3.82, *p* = 0.015), and number of affected vertebrae (OR 0.53, 95% CI 0.30–0.94, *p* = 0.031) maintained significance. The effect of the variable “visceral metastases” was removed during backward elimination with *p* = 0.14 (Wald Chi-square test).

**Table 1:** Associations between investigated factors and successful treatment (improved motor function or remaining ambulatory without aid)

Factor	Subgroup (n)	Successful treatment, n (%)	*p* value
Age	≤ 65 years (275)	35 (13)	0.47
	> 65 years (270)	29 (11)	
Interval FD to MSCC	≤ 15 months (383)	42 (11)	0.39
	> 15 months (162)	22 (14)	
Visceral metastases	No (114)	19 (17)	0.066
	Yes (431)	45 (10)	
Further bone metastases	No (126)	13 (10)	0.57
	Yes (419)	51 (12)	
Primary tumor type	Breast cancer (57)	6 (11)	**< 0.0001**
	Prostate cancer (74)	6 (8)	
	Myeloma/lymphoma (24)	10 (42)	
	Lung cancer (175)	25 (14)	
	Other malignancies (215)	17 (8)	
Sex	Female (170)	21 (12)	0.77
	Male (375)	43 (11)	
Time developing motor deficits	0–7 days (309)	16 (5)	**< 0.0001**
	8–14 days (122)	14 (11)	
	> 14 days (114)	34 (30)	
Ambulatory prior to radiotherapy	No (375)	28 (7)	**< 0.0001**
	Yes (170)	36 (21)	
Number of affected vertebrae	1–2 (192)	35 (18)	**< 0.001**
	≥ 3 (353)	29 (8)	
Radiotherapy regimen	1 × 8 Gy/5 × 4 Gy (239)	22 (9)	0.10
	5 × 5 Gy/longer-course RT (306)	42 (14)	
Entire cohort	N = 545	64 (12)	

*FD* First diagnosis of malignancy, *MSCC* Metastatic spinal cord compression, *RT* Radiotherapy

*p* values were calculated with the Chi-square test or the Fisher’s exact test (for n < 5 in at least one cell). When applying Bonferroni adjustment, *p* values of < 0.005 were considered significant and are given in bold

On univariable analyses, post-treatment ambulatory status was significantly associated with favorable tumor type (myeloma/lymphoma, *p* < 0.001), time developing motor deficits > 14 days (*p* < 0.0001), pre-treatment ambulatory status (*p* < 0.0001), and affection of 1–2 vertebrae (*p* < 0.0001) (Table 2). In the multivariable analysis, primary tumor type (OR 6.35, 95% CI 1.90–21.23, *p* = 0.003), time developing motor deficits (OR 5.41, 95% CI 2.72–10.73, *p* < 0.0001), and pre-treatment ambulatory status (OR 23.55, 95% CI 12.51–44.36, *p* < 0.0001) were significant. The effect of the variable “number of affected vertebrae” was removed during backward elimination with *p* = 0.28.

**Table 2:** Associations between investigated factors and post-treatment ambulatory status

Factor	Subgroup (n)	Ambulatory after treatment, n (%)	*p* value
Age	≤ 65 years (275)	71 (26)	0.90
	> 65 years (270)	71 (26)	
Interval FD to MSCC	≤ 15 months (383)	99 (26)	0.87
	> 15 months (162)	43 (27)	
Visceral metastases	No (114)	29 (25)	0.87
	Yes (431)	113 (26)	
Further bone metastases	No (126)	35 (28)	0.62
	Yes (419)	107 (26)	
Primary tumor type	Breast cancer (57)	16 (28)	**< 0.001**
	Prostate cancer (74)	9 (12)	
	Myeloma/lymphoma (24)	13 (54)	
	Lung cancer (175)	54 (31)	
	Other malignancies (215)	50 (23)	
Sex	Female (170)	48 (28)	0.43
	Male (375)	94 (25)	
Time developing motor deficits	0–7 days (309)	40 (13)	**< 0.0001**
	8–14 days (122)	39 (32)	
	> 14 days (114)	63 (55)	
Ambulatory prior to radiotherapy	No (375)	24 (6)	**< 0.0001**
	Yes (170)	118 (69)	
Number of affected vertebrae	1–2 (192)	70 (36)	**< 0.0001**
	≥ 3 (353)	72 (20)	
Radiotherapy regimen	1 × 8 Gy/5 × 4 Gy (239)	9 (16)	0.40
	5 × 5 Gy/longer-course RT (306)	133 (27)	
Entire cohort	N = 545	142 (26)	

*FD* First diagnosis of malignancy, *MSCC* Metastatic spinal cord compression, *RT* Radiotherapy

*p* values were calculated with the Chi-square test. When applying Bonferroni adjustment, *p* values of < 0.005 were considered significant and are given in bold

In the 375 non-ambulatory patients, regaining the ability to walk was significantly associated with favorable tumor type (myeloma/lymphoma, *p* < 0.001), time developing motor deficits > 14 days (*p* < 0.0001), and affection of 1–2 vertebrae (*p* = 0.028) (Table 3). In the multivariable analysis, primary tumor type (OR 7.35, 95% CI 1.82–29.65, *p* = 0.042) and time developing motor deficits (OR 7.45, 95% CI 2.61–21.24, *p* < 0.001) remained significant. The effect of the variable “number of affected vertebrae” was removed during backward elimination with *p* = 0.16.

**Table 3:** Associations between investigated factors and regain of ambulatory status after treatment

Factor	Subgroup (n)	Regaining ambulatory status, n (%)	*p* value
Age	≤ 65 years (190)	11 (6)	0.62
	> 65 years (185)	13 (7)	
Interval FD to MSCC	≤ 15 months (261)	15 (6)	0.43
	> 15 months (114)	9 (8)	
Visceral metastases	No (90)	9 (10)	0.11
	Yes (285)	15 (5)	
Further bone metastases	No (78)	4 (5)	0.80
	Yes (297)	20 (7)	
Primary tumor type	Breast cancer (31)	1 (3)	**< 0.001**
	Prostate cancer (61)	3 (5)	
	Myeloma/lymphoma (15)	5 (33)	
	Lung cancer (116)	8 (7)	
	Other malignancies (152)	7 (5)	
Sex	Female (112)	9 (8)	0.40
	Male (263)	15 (6)	
Time developing motor deficits	0–7 days (251)	7 (3)	**< 0.0001**
	8–14 days (71)	7 (10)	
	> 14 days (53)	10 (19)	
ECOG performance score	1–2 (4)	1 (25)	0.23
	3–4 (371)	23 (6)	
Number of affected vertebrae	1–2 (113)	12 (11)	0.028
	≥ 3 (262)	12 (5)	
Radiotherapy regimen	1 × 8 Gy/5 × 4 Gy (168)	8 (5)	0.24
	5 × 5 Gy/longer-course RT (207)	16 (8)	
Entire cohort	N = 375	24 (6)	

*FD* First diagnosis of malignancy, *MSCC* Metastatic spinal cord compression, *ECOG* Eastern Cooperative Oncology Group, *RT* Radiotherapy

*p* values were calculated with the Chi-square test or the Fisher’s exact test (for n < 5). When applying Bonferroni adjustment, *p* values of < 0.005 were considered significant and are given in bold

In the 170 ambulatory patients, maintaining the ability to walk was significantly associated with time developing motor deficits > 14 days (*p* < 0.001), and ECOG performance score of 1–2 (*p* < 0.001) (Table 4). A trend was found for favorable tumor type (myeloma/lymphoma, *p* = 0.069). In the multivariable analysis, time developing motor deficits (OR 5.18, 95% CI 1.94–13.85, *p* = 0.004) and ECOG performance score (OR 0.27, 95% CI 0.12–0.63, *p* = 0.003) achieved significance, and primary tumor type (OR 3.74, 95% CI 0.40–35.32, *p* = 0.053) showed a strong trend.

**Table 4:** Associations between investigated factors and maintainance of ambulatory status after treatment

Factor	Subgroup (n)	Maintaining ambulatory status, n (%)	*p* value
Age	≤ 65 years (85)	60 (71)	0.74
	> 65 years (85)	58 (68)	
Interval FD to MSCC	≤ 15 months (122)	84 (69)	0.80
	> 15 months (48)	34 (71)	
Visceral metastases	No (24)	20 (83)	0.15
	Yes (146)	98 (67)	
Further bone metastases	No (48)	31 (65)	0.39
	Yes (122)	87 (71)	
Primary tumor type	Breast cancer (26)	15 (58)	0.069
	Prostate cancer (13)	6 (46)	
	Myeloma/lymphoma (9)	8 (89)	
	Lung cancer (59)	46 (78)	
	Other malignancies (63)	43 (68)	
Sex	Female (58)	39 (67)	0.66
	Male (112)	79 (71)	
Time developing motor deficits	0–7 days (58)	33 (57)	**< 0.001**
	8–14 days (51)	32 (63)	
	> 14 days (61)	53 (87)	
ECOG performance score	1–2 (69)	59 (86)	**< 0.001**
	3–4 (101)	59 (58)	
Number of affected vertebrae	1–2 (79)	58 (73)	0.29
	≥ 3 (91)	60 (66)	
Radiotherapy regimen	1 × 8 Gy/5 × 4 Gy (71)	50 (70)	0.81
	5 × 5 Gy/Longer-course RT (99)	68 (69)	
Entire cohort	N = 170	118 (69)	

*FD* First diagnosis of malignancy, *MSCC* Metastatic spinal cord compression, *ECOG* Eastern Cooperative Oncology Group, *RT* Radiotherapy

*p* values were calculated with the Chi-square test or the Fisher’s exact test (for n < 5). When applying Bonferroni adjustment, *p* values of < 0.005 were considered significant and are given in bold

In addition, patients who died ≤ 1 month (n = 220) were compared to those patients who died > 1 month following radiotherapy (n = 325) with respect to successful treatment, post-treatment ambulatory status, regain of ambulatory status, and maintenance of ambulatory status. Treatment was successful in 32 of 220 patients (15%) and 32 of 325 patients (10%), respectively (*p* = 0.095); 57 (26%) and 85 patients (26%), respectively, were ambulatory following radiotherapy (*p* = 0.95). Fourteen of 156 patients (9%) and 10 of 219 patients (5%), respectively, regained the ability to walk (*p* = 0.086), and 43 of 64 patients (67%) and 75 of 106 patients (71%), respectively, maintained their ambulatory status (*p* = 0.62).

The three factors significantly associated on multivariable analyses with both successful treatment and post-treatment ambulatory status (primary tumor type, time developing motor deficits, pre-treatment ambulatory status) were used for creating the scoring tool. Scoring points ranged between 0 and 3 points (Table 5). Based on treatment success and post-treatment ambulatory rates (Table 6), three groups were designed, namely 0, 1 and 2–3 points. Treatment success rates were 4%, 15% and 39%, respectively (*p* < 0.0001), and post-treatment ambulatory rates were 4%, 43% and 86%, respectively (*p* < 0.0001).

**Table 5:** Pre-treatment factors significantly associated with both successful treatment and post-treatment ambulatory status and corresponding scoring points

Factor	Subgroup (n)	Scoring points
Primary tumor type	Breast cancer (57)	0
	Prostate cancer (74)	0
	Lung cancer (175)	0
	Other malignancies (215)	0
	Myeloma/lymphoma (24)	1
Time developing motor deficits	0–7 days (309)	0
	8–14 days (122)	0
	> 14 days (114)	1
Ambulatory prior to radiotherapy	No (375)	0
	Yes (170)	1

**Table 6:** Treatment success and post-treatment ambulatory ststus of the three prognostic groups

Prognostic group	Successful treatment (*p* < 0.0001)	Ambulatory post-treatment (*p* < 0.0001)	Regaining ambulatory status (*p* < 0.0001)	Maintaining ambulatory status (*p* < 0.0001)
0 Points	4% (13/311)	4% (11/311)	4% (11/311)	Not available
1 Point	15% (24/164)	43% (71/164)	18% (11/60)	58% (60/104)
2–3 Points	39% (27/70)	86% (60/70)	50% (2/4)	58% (58/66)

## Discussion

Personalization of the treatment is very important for patients with MSCC. Selected patients with an expected survival time of a least 3 months can benefit from upfront decompressive surgery in addition to radiotherapy. In a small randomized trial (n = 101), the combined treatment resulted in a significantly higher post-treatment ambulatory rate than radiotherapy alone (84% vs. 57%, *p* = 0.001) [3]. Moreover, a higher proportion of non-ambulatory patients regained the ability to walk (62% vs. 19%, *p* = 0.01). However, many patients with MSCC have survival prognoses of less than 3 months and are not considered candidates for upfront surgery. For patients with very limited survival prognoses, BSC alone was considered reasonable in several studies [4, 10, 11]. Identification of these patients can be facilitated with survival scores that are available for palliative radiotherapy in general [12–15] and for patients with MSCC [4]. In a retrospective study evaluating 33 patients (94% with brain, bone or lung metastases), who died within 30 days after palliative radiotherapy, about half of the patients did not benefit from radiotherapy but spent most of their remaining lifespan on therapy [11]. In another retrospective study, 54 patients died within 30 days after radiotherapy [12]. Radiotherapy in the last month of life provided only minimal palliation. In a larger retrospective study BSC alone was recommended for patients with a high probability to die within 2 months [4]. Depending on the symptoms to be controlled, BSC should include corticosteroids and analgesics.

However, in a retrospective study of 232 patients who died within 3 months after the start of radiotherapy for symptomatic bone metastases, pain relief was observed in 70% of patients at 1 month and 63% of patients at 2 months, respectively [16]. Therefore, the authors suggested that patients with painful bone metastases should be considered for palliative radiotherapy despite their limited remaining lifespan. Moreover, since radiotherapy is generally quite effective regarding preservation or improvement of motor function, also patients close to end of life may benefit from palliative irradiation. To identify these patients, a scoring tool was developed in the present study. Main endpoints included successful treatment and post-treatment ambulatory rate. Three factors were found to be independent predictors with respect to both endpoints, namely primary tumor type, time developing motor deficits prior to radiotherapy and pre-treatment ambulatory status. Myeloma and lymphomas causing spinal cord compression were previously reported to be associated with high response rates after radiotherapy. In a retrospective study of 238 patients with spinal cord compression from vertebral myeloma, 53% of the patients showed improvement of motor function and 88% were able to walk following radiotherapy alone [17]. In another retrospective study of 29 patients with spinal cord compression caused by vertebral lymphoma, improvement and post-treatment ambulatory (after 1 month) rates were 72% and 83%, respectively [18]. After 6 months, 100% of the patients alive were able to walk. The fact that a time developing motor deficits prior to radiotherapy > 14 days (representing a slower development of the deficits) was associated with higher response rates was also previously reported. In a prospective non-randomized study of 98 patients irradiated for MSCC, improvement of motor deficits occurred significantly more often if motor deficits developed > 14 days compared to 8–14 days and ≤ 7 days (86% vs. 29% and 10%, *p* < 0.001) [19]. The post-treatment ambulatory rate was also significantly higher (86% vs. 55% and 35%, *p* = 0.026). Moreover, in a large retrospective study of 1,304 patients, corresponding rates of improvement of motor function were 41%, 24% and 7%, respectively (*p* < 0.001) [7]. These findings can be explained by differences regarding the decrease in arterial and venous blood flow. Acute deterioration of motor function may be the consequence of disruption of the arterial circulation leading to spinal cord infarction, whereas more slowly developing motor deficits are considered a consequence of venous congestion, which is more likely to be reversible [19, 20]. The prognostic role of the pre-treatment ambulatory status with respect to improvement of motor function was also reported before [7, 21].

Based on these three independent predictors of outcome (primary tumor type, time developing motor deficits prior to radiotherapy and pre-treatment ambulatory status), the prognostic score was developed that included three groups, namely 0 points, 1 point and 2–3 points. In the 0-points group, treatment success and post-treatment ambulatory rates were only 4% and 4%, respectively. Moreover, only 4% of the non-ambulatory patients regained the ability to walk. Therefore, these patients did not appear to benefit from radiotherapy. This applied also to the majority of patients in the 1-point group, since the treatment success rate was only 15% and 18% of non-ambulatory patients regained ambulatory status. However, in 58% of the ambulatory patients in this group, ambulatory status was preserved. The most favorable results were found in patients of the 2–3-points group. Treatment success and post-treatment ambulatory rates were 39% and 86%, respectively. Moreover, two of the four non-ambulatory patients (50%) became ambulatory after radiotherapy, and 88% of the 66 ambulatory patients maintained their gait function. Therefore, these patients did benefit from radiotherapy and should receive palliative radiation treatment, preferably with 1 × 8 Gy or 5 × 4 Gy. These regimens appeared not inferior to other regimens with respect to the investigated endpoints. These findings agree with the results of the large retrospective study of 1,304 patients, where 1 × 8 Gy and 5 × 4 Gy resulted in similar effects on motor deficits and post-treatment ambulatory rates when compared to 10 × 3 Gy, 15 × 2.5 Gy and 20 × 2 Gy [7]. Moreover, in a randomized phase III trial of 203 patients with poor or intermediate survival prognoses, no significant differences were found between 5 × 4 Gy and 10 × 3 Gy with respect to effects on motor deficits and post-treatment ambulatory rates [22]. When interpreting the results of the present study and aiming to utilize the new score, the retrospective nature of the data used to create the score and the risk of hidden selection biases should be considered.

In summary, this new score helps identify patients with MSCC who can benefit from palliative radiotherapy, although they are near end of life. In patients with 2–3 points, the rates of treatment success and post-treatment ambulatory status, as well as regaining and maintaining the ability to walk were comparably high. Therefore, these patients should receive palliative radiotherapy, preferably with very short courses such as 1 × 8 Gy or 5 × 4 Gy.

## Data Availability

The data analysed for this paper cannot be shared on a publicly available repository due to data protection regulations. According to the local ethics committee, only the evaluation of anonymized data is allowed for this study.

## References

[1] Prasad D, Schiff D (2005). Malignant spinal cord compression. Lancet Oncol.

[2] Lawton AJ, Lee KA, Cheville AL, Ferrone ML, Rades D, Balboni TA, Abrahm JL (2019). Assessment and management of patients with metastatic spinal cord compression: a multidisciplinary review. J Clin Oncol.

[3] Patchell R, Tibbs PA, Regine WF, Payne R, Saris S, Kryscio RJ, Mohiuddin M, Young B (2005). Direct decompressive surgical resection in the treatment of spinal cord compression caused by metastatic cancer: a randomised trial. Lancet.

[4] Rades D, Hueppe M, Schild SE (2013). A score to identify patients with metastatic spinal cord compression who may be candidates for best supportive care. Cancer.

[5] Tomita T, Galicich JH, Sundaresan N (1983). Radiation therapy for spinal epidural metastases with complete block. Acta Radiol Oncol.

[6] Rades D, Conde-Moreno AJ, Cacicedo J, Veninga T, Segedin B, Stanic K, Rudat V, Schild SE (2018). 1×8 Gy versus 5×4 Gy for metastatic epidural spinal cord compression: a matched-pair study of three prognostic patient subgroups. Radiat Oncol.

[7] Rades D, Stalpers LJ, Veninga T, Schulte R, Hoskin PJ, Obralic N, Bajrovic A, Rudat V, Schwarz R, Hulshof MC, Poortmans P, Schild SE (2005). Evaluation of five radiation schedules and prognostic factors for metastatic spinal cord compression. J Clin Oncol.

[8] Barendsen GW (1982). Dose fractionation, dose rate and iso-effect relationships for normal tissue responses. Int J Radiat Oncol Biol Phys.

[9] Joiner MC, Van der Kogel AJ, Steel GG (1997). The linear-quadratic approach to fractionation and calculation of isoeffect relationships. Basic clinical radiobiology.

[10] Gripp S, Mjartan S, Boelke E, Willers R (2010). Palliative radiotherapy tailored to life expectancy in end-stage cancer patients: Reality or myth?. Cancer.

[11] Patel A, Dunmore-Griffith J, Lutz S, Johnstone PA (2013). Radiation therapy in the last month of life. Rep Pract Oncol Radiother.

[12] Lee SF, Luk H, Wong A, Ng CK, Wong FCS, Luque-Fernandez MA (2020). Prediction model for short-term mortality after palliative radiotherapy for patients having advanced cancer: a cohort study from routine electronic medical data. Sci Rep.

[13] Vázquez M, Altabas M, Moreno DC, Geng AA, Pérez-Hoyos S, Giralt J (2021). 30-day mortality following palliative radiotherapy. Front Oncol.

[14] Wu SY, Singer L, Boreta L, Garcia MA, Fogh SE, Braunstein SE (2019). Palliative radiotherapy near the end of life. BMC Palliat Care.

[15] Wu SY, Yee E, Vasudevan HN, Fogh SE, Boreta L, Braunstein SE, Hong JC (2021). Risk stratification for imminent risk of death at the time of palliative radiotherapy consultation. JAMA Netw Open.

[16] Dennis K, Wong K, Zhang L, Culleton S, Nguyen J, Holden L, Jon F, Tsao M, Danjoux C, Barnes E, Sahgal A, Zeng L, Koo K, Chow E (2011). Palliative radiotherapy for bone metastases in the last 3 months of life: worthwhile or futile?. Clin Oncol (R Coll Radiol).

[17] Rades D, Conde-Moreno AJ, Cacicedo J, Segedin B, Rudat V, Schild SE (2016). Excellent outcomes after radiotherapy alone for malignant spinal cord compression from myeloma. Radiol Oncol.

[18] Rades D, Conde-Moreno AJ, Cacicedo J, Šegedin B, Rudat V, Schild SE (2016). Radiation therapy alone provides excellent outcomes for spinal cord compression from vertebral lymphoma. Anticancer Res.

[19] Rades D, Heidenreich F, Karstens JH (2002). Final results of a prospective study of the prognostic value of the time to develop motor deficits before irradiation in metastatic spinal cord compression. Int J Radiat Oncol Biol Phys.

[20] Asdourian PL, Bridwell KH, DeWald RL (1997). Natural history of metastatic spinal disease. Textbook of spinal surgery.

[21] Rades D, Lange M, Veninga T, Stalpers LJ, Bajrovic A, Adamietz IA, Rudat V, Schild SE (2011). Final results of a prospective study comparing the local control of short-course and long-course radiotherapy for metastatic spinal cord compression. Int J Radiat Oncol Biol Phys.

[22] Rades D, Šegedin B, Conde-Moreno AJ, Garcia R, Perpar A, Metz M, Badakhshi H, Schreiber A, Nitsche M, Hipp P, Schulze W, Adamietz IA, Norkus D, Rudat V, Cacicedo J, Schild SE (2016). Radiotherapy with 4 Gy × 5 versus 3 Gy × 10 for metastatic epidural spinal cord compression: final results of the SCORE-2 trial (ARO 2009/01). J Clin Oncol.

